# DLC1 deficiency at diagnosis predicts poor prognosis in acute myeloid leukemia

**DOI:** 10.1186/s40164-022-00335-5

**Published:** 2022-10-18

**Authors:** Xueqian Li, Jiaqian Qi, Xiaofei Song, Xiaoyan Xu, Tingting Pan, Hong Wang, Jingyi Yang, Yue Han

**Affiliations:** 1 National Clinical Research Center for Hematologic Diseases, Jiangsu Institute of Hematology, The First Affiliated Hospital of Soochow University, 188 Shizi Street, Suzhou, 215006 Jiangsu People’s Republic of China; 2 Institute of Blood and Marrow Transplantation, Collaborative Innovation Center of Hematology, Soochow University, Suzhou, China; 3 Key Laboratory of Thrombosis and Hemostasis of Ministry of Health, Suzhou, China; 4 State Key Laboratory of Radiation Medicine and Protection, Soochow University, Suzhou, China

**Keywords:** Acute myeloid leukemia, Weighted gene co-expression network analysis, Differential gene expression analysis, DLC1, Machine learning

## Abstract

Acute myeloid leukemia (AML) is a complex, heterogeneous malignant hematologic disease. Although multiple prognostic-related genes gave been explored in previous studies, there are still many genes whose prognostic value remains unclear. In this study, a total of 1532 AML patients from three GEO databases were included, five genes with potential prognostic value (DLC1, NF1B, DENND5B, TANC2 and ELAVL4) were screened by weighted gene co-expression network analysis (WGCNA), least absolute shrinkage and selection operator (LASSO) and support vector machine recursive feature elimination (SVM-RFE). Based on this, we conducted survival analysis of the above five genes through the TCGA database and found that low level of DLC1 was detrimental to the long-term prognosis of AML patients. We also performed external validation in 48 AML patients from our medical center to analyze the impact of DLC1 level on prognosis. In conclusion, DLC1 may be a potential marker affecting the prognosis of AML, and its deficiency is associated with poor prognosis.

**Supplementary Information:**

The online version contains supplementary material available at 10.1186/s40164-022-00335-5.

**To the Editor,**

Acute myeloid leukemia (AML) is a heterogeneous hematological malignancy characterized by malignant proliferation and abnormal differentiation of myeloid blasts [1]. The prognosis of AML remains poor, with 5-year overall survival rate below 50% after diagnosis and 2-year survival rate of only 20%–30% in elderly patients [2, 3]. Currently, molecular and genetic advances have increased our understanding of the pathogenesis and progression of AML [4, 5], and further discovery of genes associated with prognosis can broaden therapeutic options for AML patients. This study aimed to identify novel prognostic genes for AML through a multi-perspective and multi-dimensional analysis of the GEO database and validation using the TCGA database and our center's data.

As shown in the workflow (Fig. 1A), three datasets with survival outcomes were downloaded from the GEO databases, and 1532 patients were included for follow-up analysis (562 in GSE37642, 534 in GSE76009, and 436 in GSE16432). A total of 151 AML patients with RNA-seq data were enrolled from the TCGA database. Five hundred and five differentially expressed genes (DEGs) in the GEO datasets were screened using |logFC|≥ 1.0 and adj. p-value < 0.05 as cut-off criteria. The heat map shows 50 genes with the highest variability (Fig. 1B), and the volcano map displays the up-and down-regulated genes among these differential genes (Fig. 1C). Towards a comprehensive understanding of the potential function of these genes in the co-expression modules, the Gene Ontology (GO) enrichment analysis was performed and divided genes into three categories: biological processes (BP), cellular components (CC), and molecular functions (MF) (Fig. 1D). In BP, genes were enriched in GTPase activity and Golgi vesicle transport. Many genes are involved in the cell leading edge, transport vesicle, and transcription regulator complex in the CC category. In addition, protein serine/threonine kinase activity and Ras GTPase binding were mainly involved in MF. Kyoto Encyclopedia of Genes and Genomes (KEGG) enrichment analysis showed that genes were enriched in choline metabolism in cancer (Fig. 1E).

**Fig. 1:**
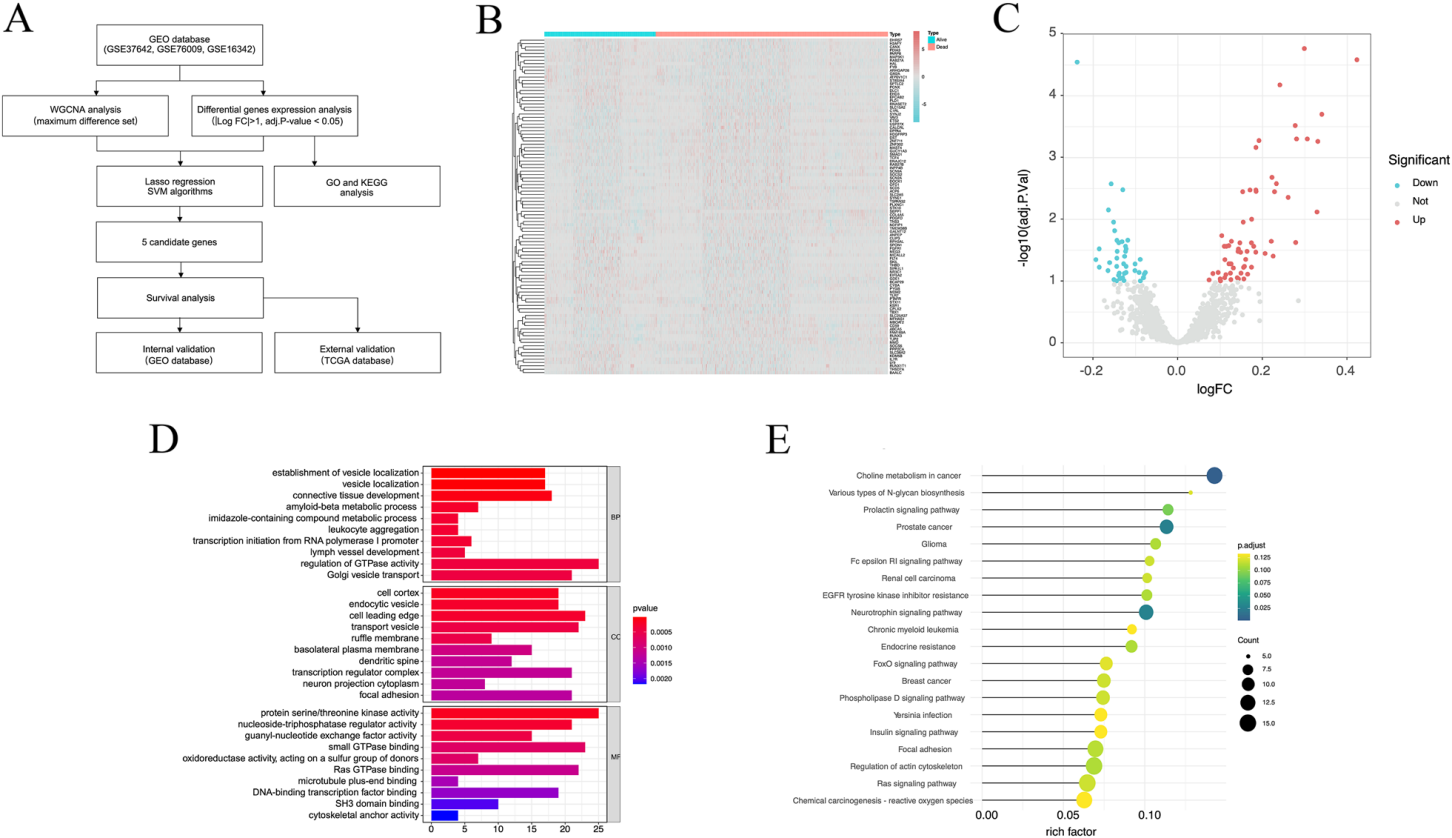
The flow-chart of this study and functional enrichment analysis of differential genes. **A** Flow chart of our study. **B** Heat map for the analysis of differential genes in AML patients with different survival status. **C** Volcano map of differential expressed genes in AML patients with different survival states. **D** GO enrichment analysis. **E** KEGG enrichment analysis. *p < 0.05, **p < 0.01, ***p < 0.001. *GO* gene ontology; *KEGG* Kyoto Encyclopedia of Genes and Genomes

Two thousand three hundred and forty-one co-expressed genes were extracted from these significant modules based on weighted gene co-expression network analysis (WGCNA) [6] (Fig. 2A). We compared the status of patients (alive or dead) in different sets of genes and found the most significant degree of variability in the gray gene set (Fig. 2B). We also analyzed the correlation of gene significance and module membership in patients with different survival statuses in both gray and blue modules (Additional file 1: Fig. S1). We intersected the eligible differential genes with the genes in the WGCNA gray gene set. Finally, 22 overlapping genes were obtained at the junction of the DEGs list and the two co-expression modules (Additional file 2: Fig. S2). Then, least absolute shrinkage and selection operator (LASSO) regression screening was performed based on the partial likelihood deviation method to obtain the corresponding coefficients with an optimal log λ of −4.35, of which 21 genes were suitable for constructing the model [7] (Additional file 3: Fig. S3A, B).

**Fig. 2:**
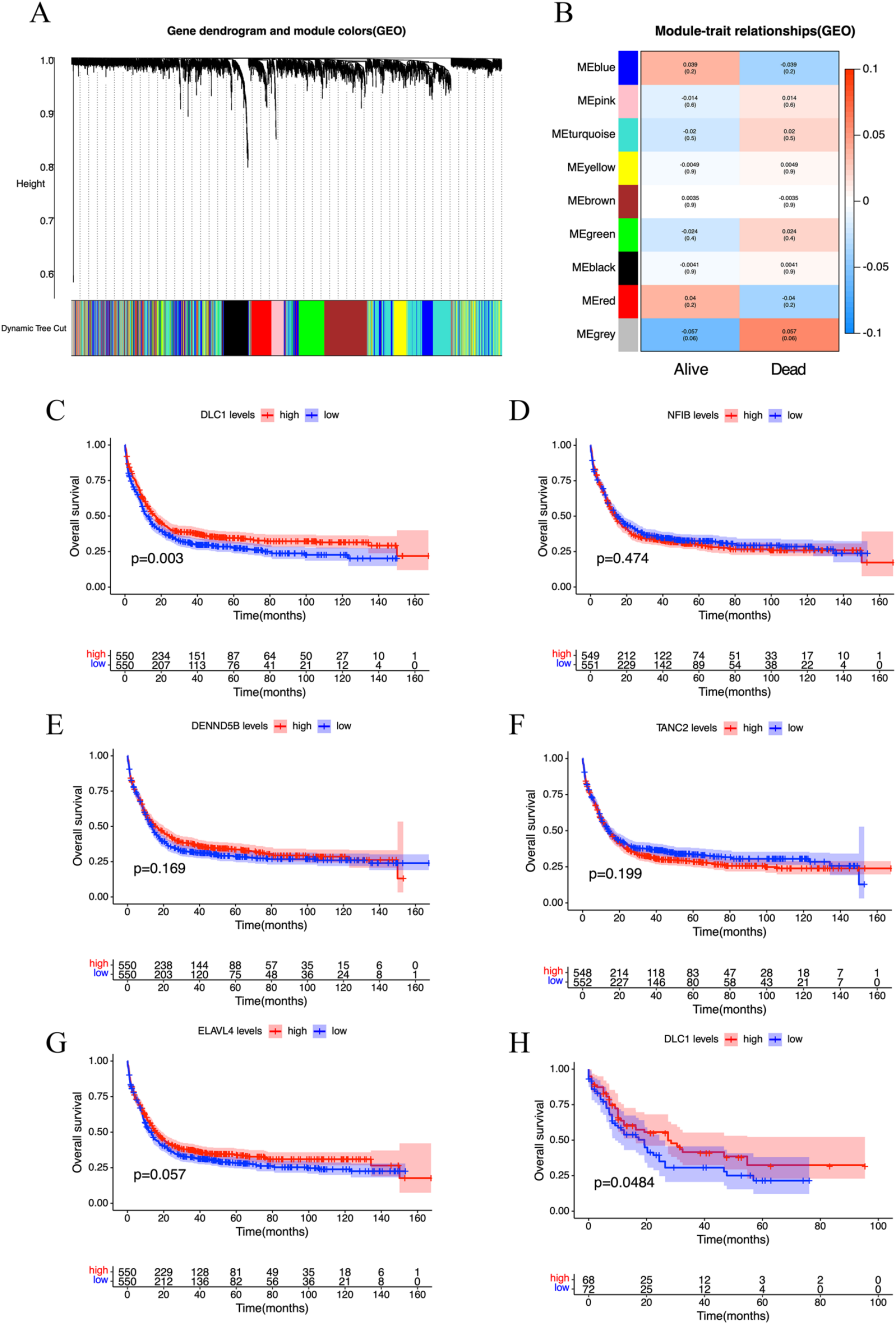
Co-expression network based on WGCNA to identify modules associated with AML survival status and survival analysis of candidate hub genes. **A** Clustering dendrogram of co-expression modules screened based on AML-related samples from the GEO database. **B** Module-trait relationships between gene modules and survival status of AML patients. **C**–**G** Kaplan–Meier survival analysis based on DLC1 (**C**), NF1B (**D**), DENND5B (**E**), TANC2 (**F**), and ELAVL4 (**G**) expression in AML patients from the GEO database. **H** Kaplan–Meier survival analysis based on DLC1 expression in AML patients from TCGA database. *p < 0.05, **p < 0.01, ***p < 0.001. *WGCNA* Weighted Gene Co-expression Network Analysis, *GEO* Gene Expression Omnibus, *TCGA* The Cancer Genome Atlas

Meanwhile, five genes were identified by the support vector machine algorithm with the optimal five CV accuracy [8] (Additional file 3: Fig. S3C). Moreover, the five shared biomarkers of AML were defined by overlapping the biomarkers derived from the two algorithms (Additional file 3: Fig. S3D). These five specific genes (DLC1, NF1B, DENND5B, TANC2, ELAVL4) are associated with the prognosis of AML. We performed a survival analysis of the above five genes using GEO data (Fig. 2C–G) and found that AML patients with low DLC1 level had a poorer overall prognosis (Fig. 2C, p = 0.003). Median was regarded as cut-off value. Survival data from TCGA data showed similar results (Fig. 2H, p = 0.048). Meanwhile, we analyzed the expression of relevant genes in patients with different survival states (Additional file 4: Fig. S4) and found that DLC1 expression was significantly lower in deceased patients (Additional file 4: Fig. S4A, *p* = 0.0005). Furthermore, Quantitative real-time PCR was used to detect DLC1 expression in bone marrow samples from 48 newly diagnosed AML patients in our center. The clinical characteristics of these patients are provided in Additional file 6: Table. S1. The Kaplan–Meier curve showed that patients with low DLC1 expression had a worse prognosis (Additional file 5: Fig. S5, *p* = 0.028).

DLC1 is a downregulated tumor suppressor gene and silenced by epigenetic mechanisms in human cancers, including several hematological malignancies [9–11]. Based on the bio-information database and external validation, our study suggests that DLC1 may be a potential marker affecting the prognosis of AML. Its deficiency at initial diagnosis is associated with a poor long-term prognosis. Future AML-matched methylation and RNA-seq are required to better elucidate the mechanism of DLC1 downregulation to guide the development of relevant molecular targeting drugs.

## Supplementary Information

**Additional file 1: Figure S1.** Correlation between gene significance and related module membership. A. The correlation between gene significance and module membership in the gray module. B. The correlation between gene significance and module membership in the blue module. *p < 0.05, **p < 0.01, ***p < 0.001.

**Additional file 2: Figure S2.** A Venn diagram shows the overlap of 22 optimal hub genes between GEO differential genes and WGCNA grey gene set.

**Additional file 3: Figure S3.** Identification of candidate hub genes to predict prognosis for AML patients. A. Partial likelihood deviance for different numbers of variables revealed by the LASSO regression model. Red dots represent the partial likelihood deviance values. Gray lines represent the partial likelihood deviance ± standard error. B. Fourteen candidate genes with minimum lambda values were obtained by LASSO regression with tenfold cross-validation. C. Candidate genes were filtrated from SVM-RFE algorithms. D. Five optimal hub genes overlapped between LASSO and SVM-RFE algorithm were shown by Venn diagram. Abbreviations: LASSO, least absolute shrinkage and selection operator; SVM-RFE, support vector machine recursive feature elimination.

**Additional file 4: Figure S4.** The expression levels of DLC1, NFIB, DENND5B, TANC2, and ELAVL4 in AML patients with different survival status. Scatter plots of the five gene’s expression level in AML patients with different survival status based on GEO dataset. *p < 0.05, **p < 0.01, ***p < 0.001. Abbreviations: GEO, Gene Expression Omnibus.

**Additional file 5: Figure S5.** Kaplan-Meier survival curve according to the DLC1 expression level for 48 AML patients in our center. Kaplan-Meier plots was used to visualize the overall survival probability of 48 patients based on the expression of DLC1 at diagnosis in our center.

**Additional file 6: Table S1.** Clinical characteristics of 48 AML patients. **Table S2.** Basic information for the included GEO databases.

## Data Availability

Original data and code generated or used during the study are available from the corresponding author if necessary.

## Abbreviations

AML: Acute myeloid leukemia
BP: Biological processes
CC: Cellular components
DEGs: Differentially expressed genes
GEO: Gene expression omnibus
GO: Gene ontology
KEGG: Kyoto encyclopedia of genes and genomes
LASSO: Least absolute shrinkage and selection operator
MF: Molecular functions
PCR: Polymerase chain reaction
SVM-RFE: Support vector machine recursive feature elimination
TCGA: The cancer genome atlas
WGCNA: Weighted gene co-expression network analysis
