# Supplementary material for: DLC1 deficiency at diagnosis predicts poor prognosis in acute myeloid leukemia
Source: Exp Hematol Oncol. 2022 Oct 18;11:74. doi: 10.1186/s40164-022-00335-5 (PMC9580124; doi:10.1186/s40164-022-00335-5)
Supplement: Supplementary file 1 — Additional file 1: Figure S1. Correlation between gene significance and related module membership. A. The correlation between gene significance and module membership in the gray module. B. The correlation between gene significance and module membership in the blue module. *p < 0.05, **p < 0.01, ***p < 0.001. [file 40164_2022_335_MOESM1_ESM.pdf]

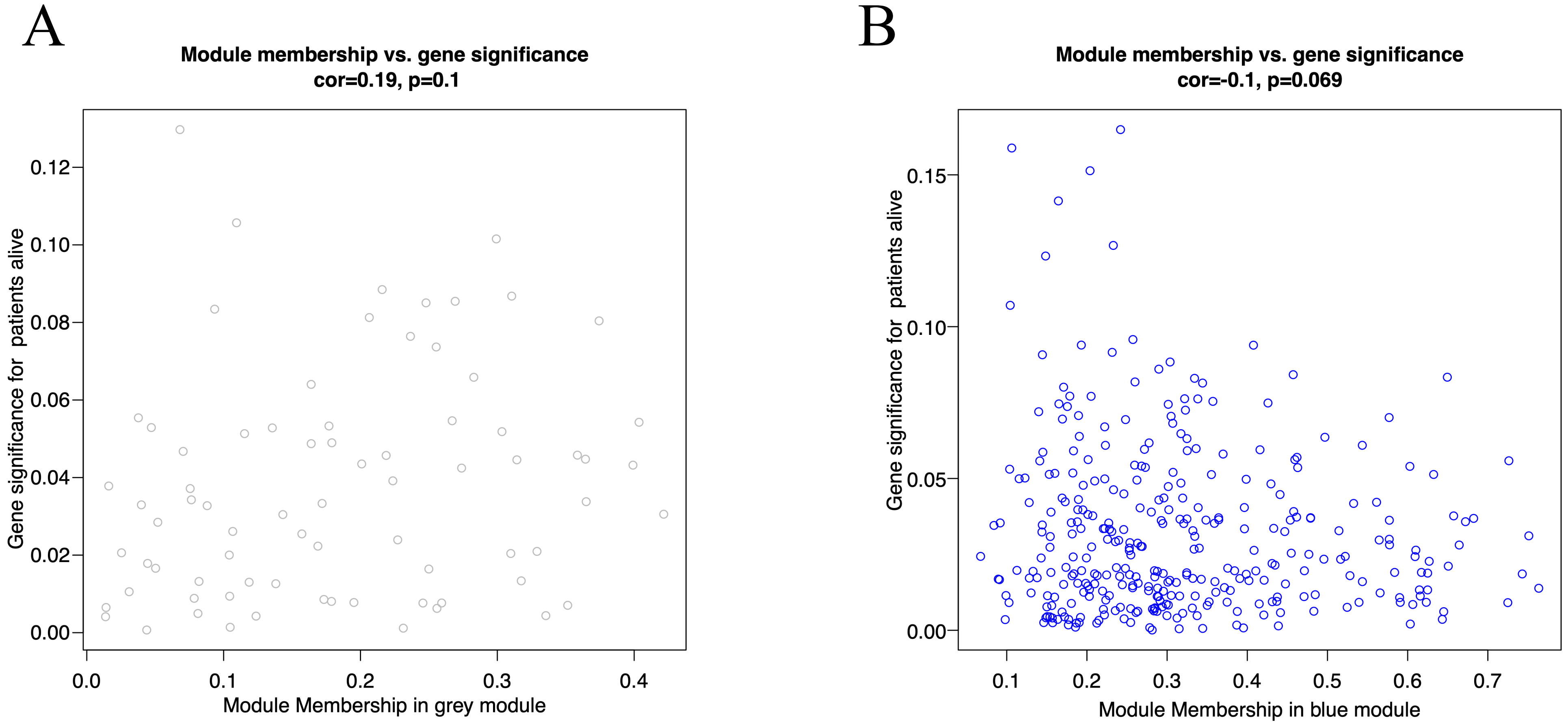
A
Module membership vs. gene significance
cor=0.19, p=0.1
Gene significance for patients alive
Module Membership in grey module
0.00
0.02
0.04
0.06
0.08
0.10
0.12
0.0
0.1
0.2
0.3
0.4
B
Module membership vs. gene significance
cor=-0.1, p=0.069
Gene significance for patients alive
Module Membership in blue module
0.00
0.05
0.10
0.15
0.1
0.2
0.3
0.4
0.5
0.6
0.7
