# Supplementary figures and images for: DLC1 deficiency at diagnosis predicts poor prognosis in acute myeloid leukemia
Source: Exp Hematol Oncol. 2022 Oct 18;11:74. doi: 10.1186/s40164-022-00335-5 (PMC9580124; doi:10.1186/s40164-022-00335-5)

GEO differential genes

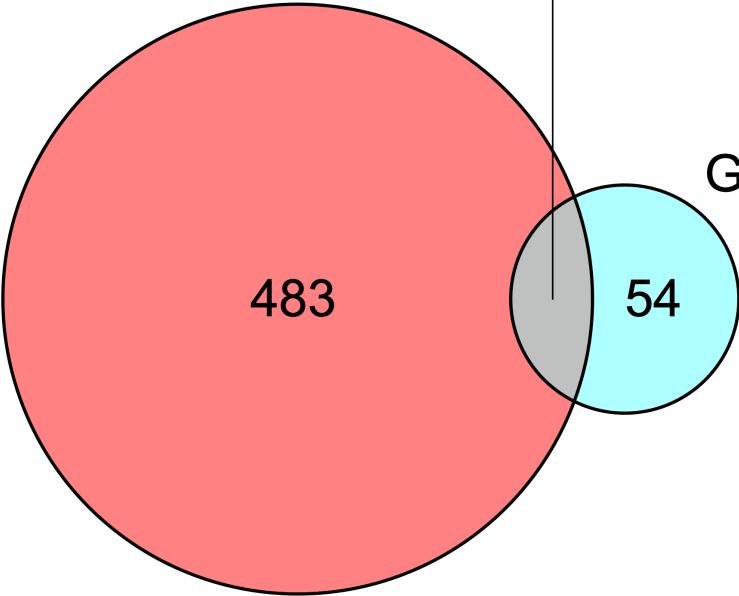

GEO WGCNA grey set

Supplement: Supplementary file 2 — Additional file 2: Figure S2. A Venn diagram shows the overlap of 22 optimal hub genes between GEO differential genes and WGCNA grey gene set. [file 40164_2022_335_MOESM2_ESM.pdf]

A

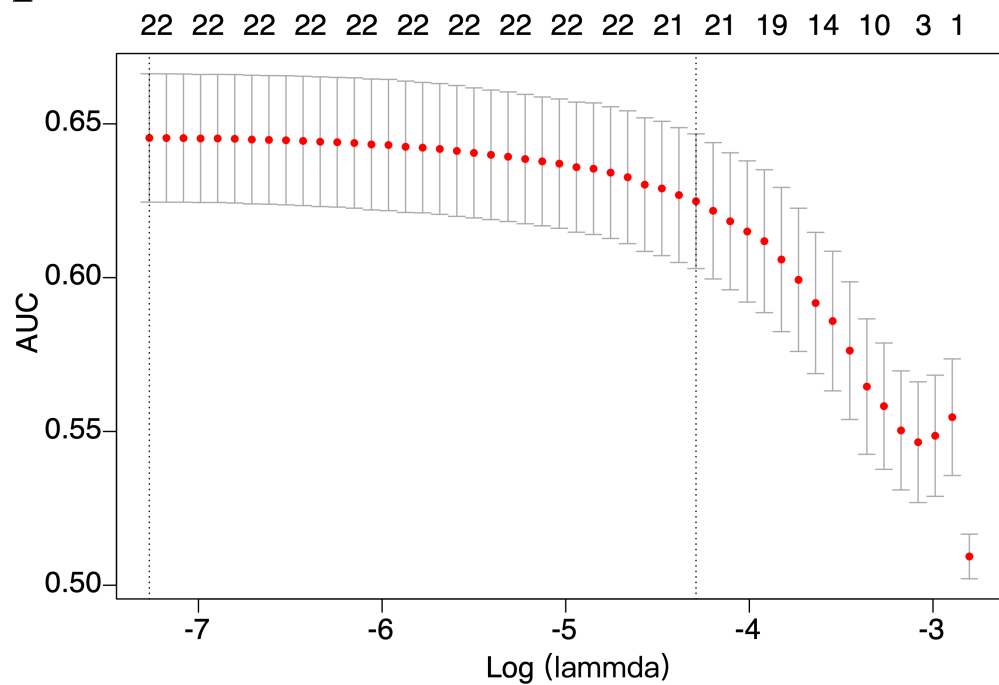

B

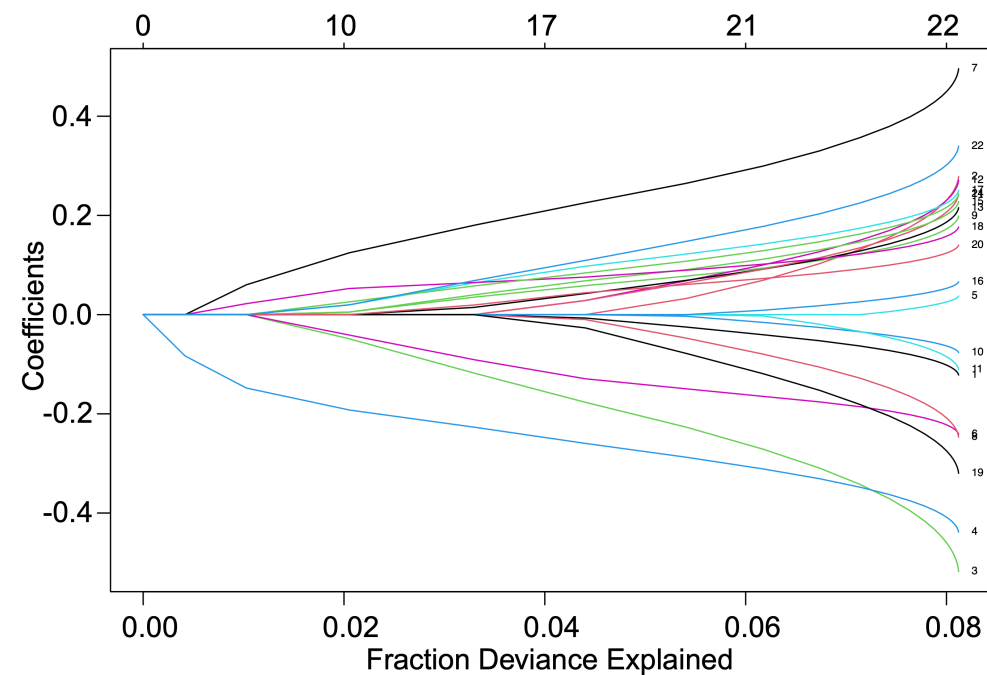

C

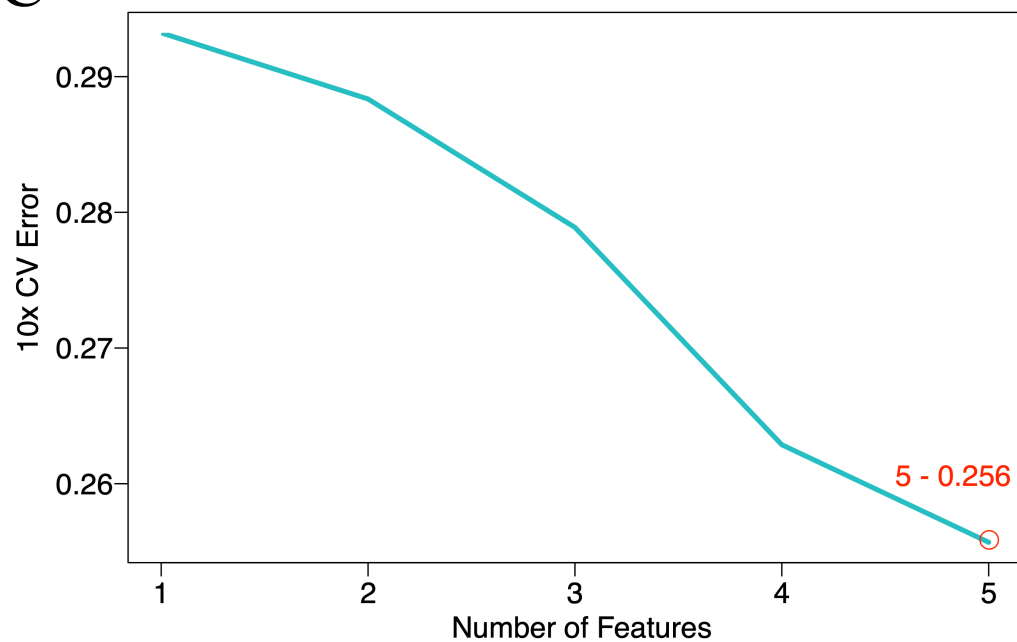

D

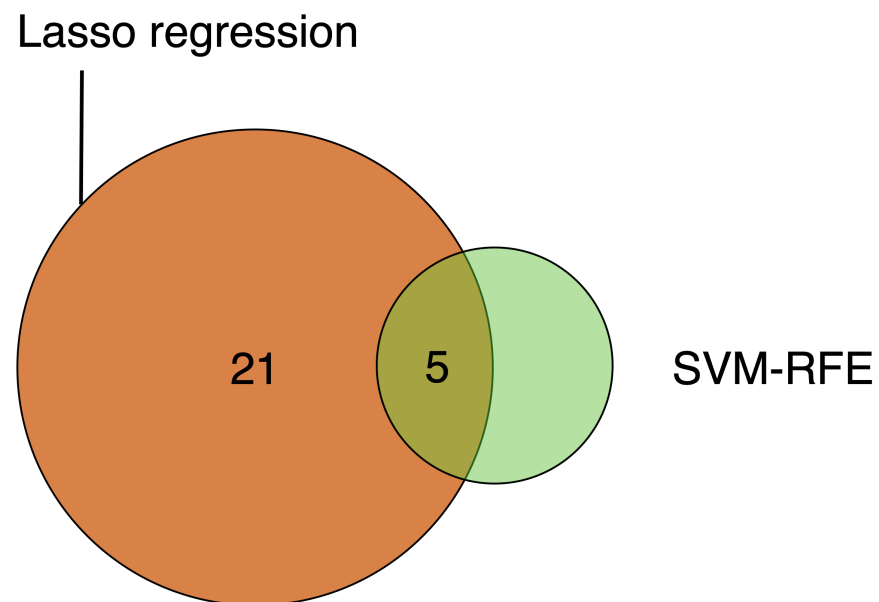

Supplement: Supplementary file 3 — Additional file 3: Figure S3. Identification of candidate hub genes to predict prognosis for AML patients. A. Partial likelihood deviance for different numbers of variables revealed by the LASSO regression model. Red dots represent the partial likelihood deviance values. Gray lines represent the partial likelihood deviance ± standard error. B. Fourteen candidate genes with minimum lambda values were obtained by LASSO regression with tenfold cross-validation. C. Candidate genes were filtrated from SVM-RFE algorithms. D. Five optimal hub genes overlapped between LASSO and SVM-RFE algorithm were shown by Venn diagram. Abbreviations: LASSO, least absolute shrinkage and selection operator; SVM-RFE, support vector machine recursive feature elimination. [file 40164_2022_335_MOESM3_ESM.pdf]

A

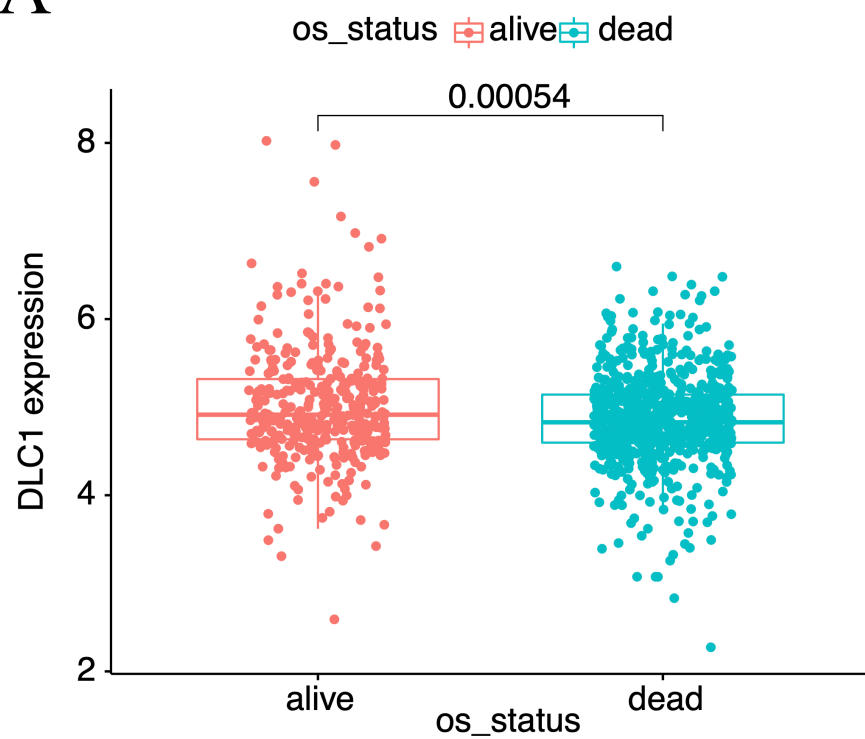

B

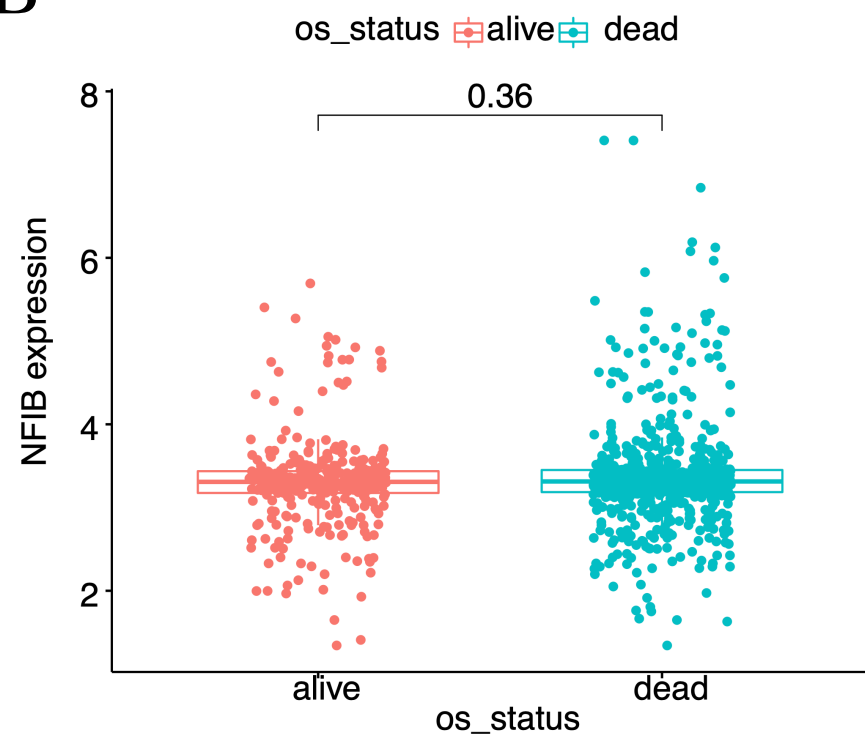

C

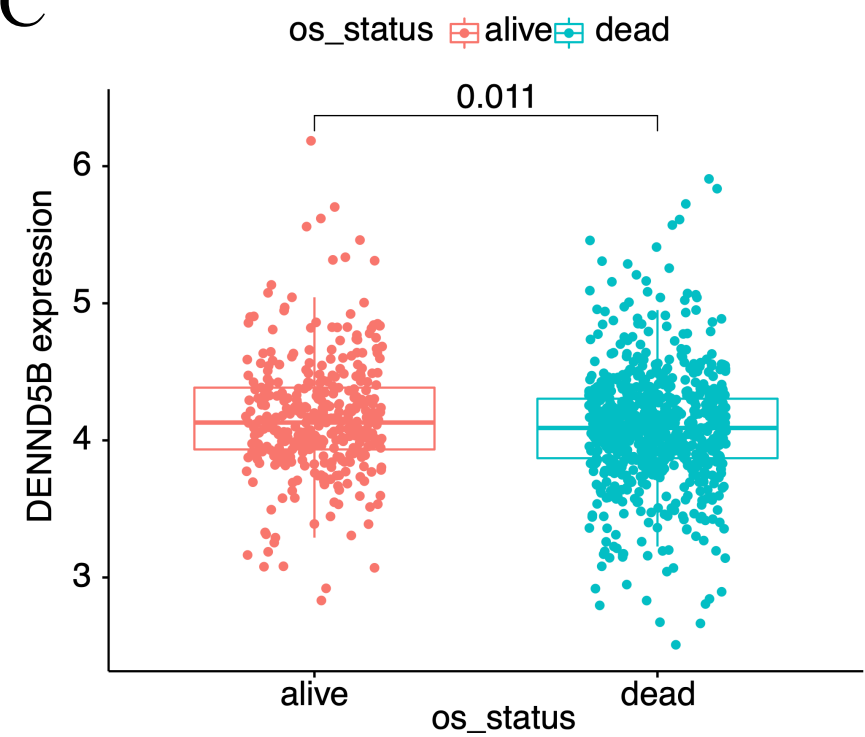

D

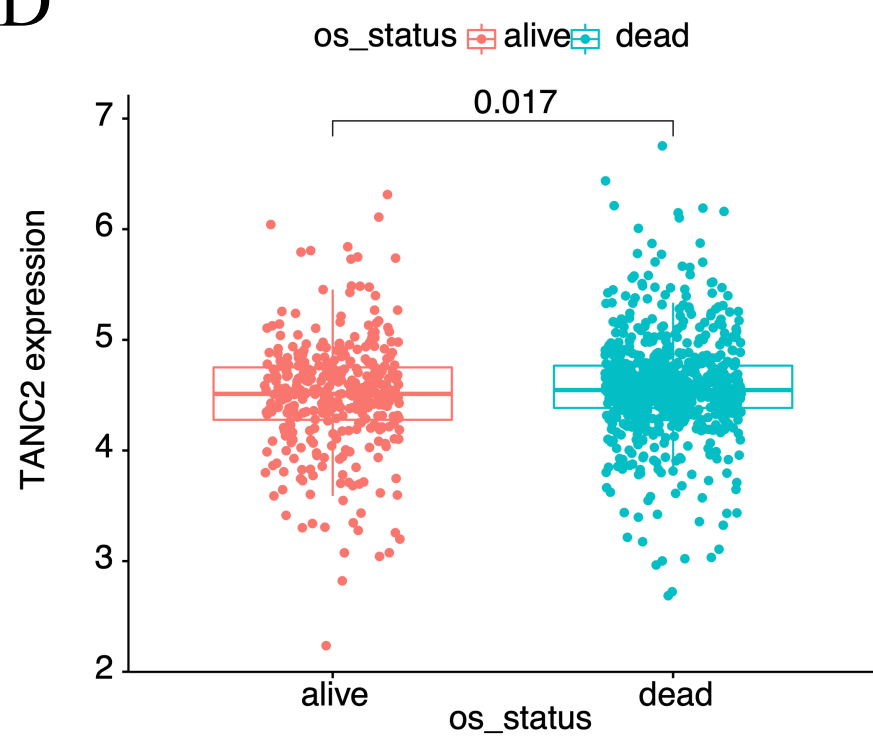

E

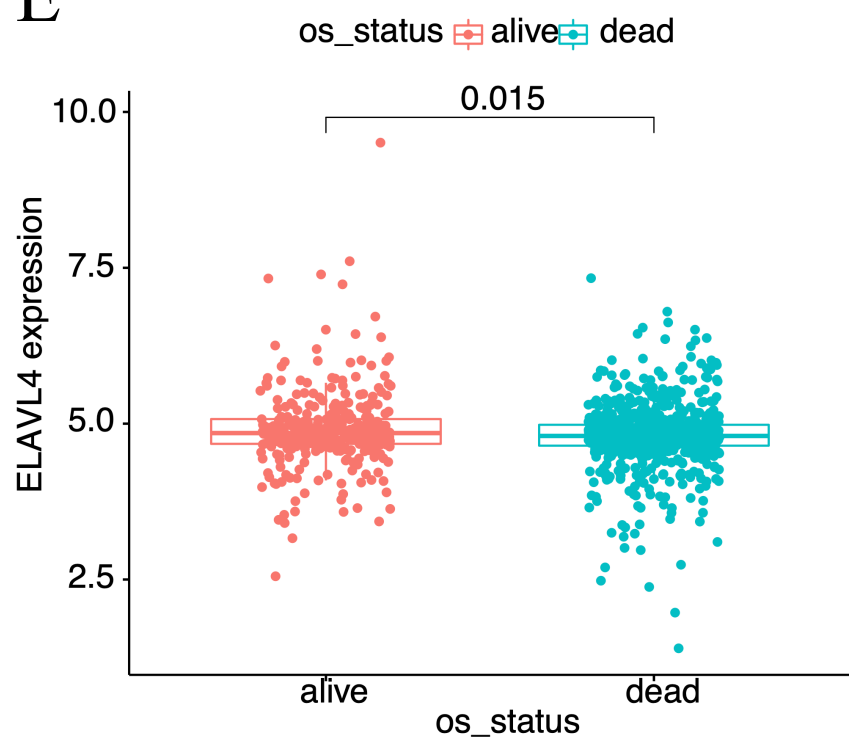

Supplement: Supplementary file 4 — Additional file 4: Figure S4. The expression levels of DLC1, NFIB, DENND5B, TANC2, and ELAVL4 in AML patients with different survival status. Scatter plots of the five gene’s expression level in AML patients with different survival status based on GEO dataset. *p < 0.05, **p < 0.01, ***p < 0.001. Abbreviations: GEO, Gene Expression Omnibus. [file 40164_2022_335_MOESM4_ESM.pdf]

Low level High level

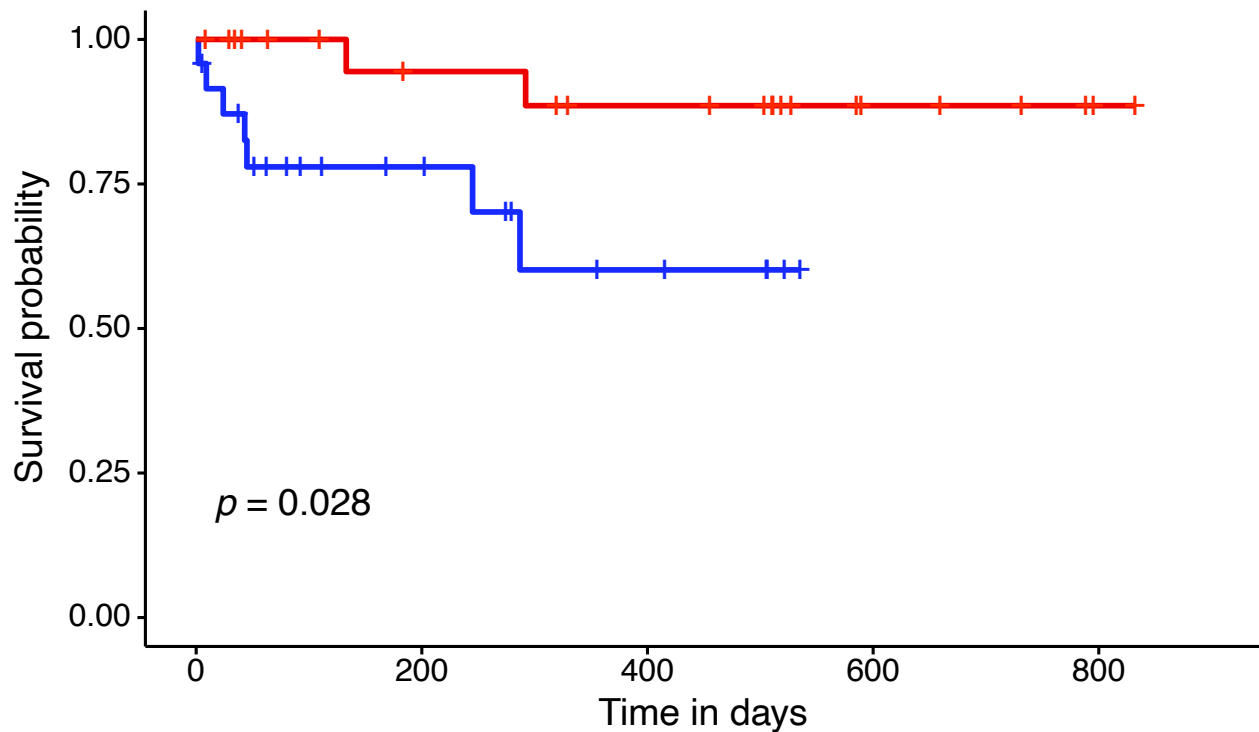

Number at risk

Low level

24

11

5

0

0

High level

24

16

13

5

1

0

200

400

600

800

Supplement: Supplementary file 5 — Additional file 5: Figure S5. Kaplan-Meier survival curve according to the DLC1 expression level for 48 AML patients in our center. Kaplan-Meier plots was used to visualize the overall survival probability of 48 patients based on the expression of DLC1 at diagnosis in our center. [file 40164_2022_335_MOESM5_ESM.pdf]
