# Supplementary material for: DLC1 deficiency at diagnosis predicts poor prognosis in acute myeloid leukemia
Source: Exp Hematol Oncol. 2022 Oct 18;11:74. doi: 10.1186/s40164-022-00335-5 (PMC9580124; doi:10.1186/s40164-022-00335-5)
Supplement: Supplementary file 6 — Additional file 6: Table S1. Clinical characteristics of 48 AML patients. Table S2. Basic information for the included GEO databases. [file 40164_2022_335_MOESM6_ESM.docx]

**DLC1 deficiency at diagnosis predicts poor prognosis in** **acute myeloid leukemia**

Xueqian Li^1,2,3,4*^, Jiaqian Qi^1,2,3,4*^, Xiaofei Song^1,2,3,4*^, Xiaoyan Xu^1,2,3,4^, Tingting Pan^1,2,3,4^, Hong Wang^1,2,3,4^, Jingyi Yang^1,2,3,4^, Yue Han^1,2,3,4#^

**Additional Data**

**Index**

1. **Additional** Text
2. **Additional** Tables
3. **Additional** Figures
4. **Additional** References
5. **Additional Text**

**Additional Methods**

**Data filtering and processing**

AML-related gene expression profiles and clinical data were downloaded from the TCGA (https://portal.gdc.cancer.gov/) and GEO (https:// www.ncbi.nlm.nih.gov/gds) database. A total of 151 acute myeloid leukemia (AML) patients with RNA-seq data were enrolled from the TCGA database, which the *TCGAbiolinks* R package downloaded [1]. As suggested by the tutorial, data filtering was performed using function *rpkm* in *edgeR* package [2].

We reduce bias between databases by combining data in two steps: data normalization and correction for batch effects. Step 1: We convert the Counts data into TPM data for data downloaded from the GEO database. The data were then normalized using the 'normalizeBetweenArrays()' function in the 'limma' package. Step 2: We define different batches for batches measured at different times in the same dataset and for data from different databases and use the 'removeBatchEffect()' function in the 'sva' package to normalize the different batches of The 'removeBatchEffect()' function in the 'sva' package is used to downgrade batches of data for further analysis [3].

After searching the GEO database, three datasets with survival outcomes were downloaded using R package *GEOquery* in this study (GSE37642, GSE76009, and GSE16432) [4]. The patients in these three datasets were as follows: 562 in GSE37642, 534 in GSE76009, and 436 in GSE16432, respectively. As a result, a total of 1100 patients containing survival data from the GEO database were included for survival analysis.

**Differential gene expression analysis**

To figure out the differentially expressed genes (DEGs) between AML patients with different survival status (alive or dead), the R package *limma* was applied in the TCGA and GEO database. The DEGs were screened with the criteria |logFC| ≥ 1.0 and adj. P-value < 0.05. The R package *ggplot2* and *VennDiagram* were used to draw a volcano map and Venn diagram [5].

**Weighted gene co-expression network analysis (WGCNA)**

To improve the accuracy of network construction, weighted gene co-expression network analysis (WGCNA) was filtered to screen genes. The R package *WGCNA* was used to conduct WGCNA between the gene expression data profiles of TCGA-LAML and GEO datasets, grouping highly co-expressed genes into modules [6]. We use *pickSoftThreshold* to establish a scale-free network. A similarity matrix was built after performing the Pearson correlation of all gene pairs. Then, the adjacency matrix was transformed into topological overlap matrix (TOM) and the TOM-based dissimilarity matrix for hierarchical clustering. We then correlated the previously computed modular features with clinical characteristics to identify the functional modules of the co-expression network.

**Function and pathway enrichment analysis**

To determine the functional relevance of these modules, we tested whether the selected genes from the modules were enriched for specific functions or signaling pathways. Gene ontology (GO) and Kyoto Encyclopedia of Genes and Genomes (KEGG) pathway analysis were performed by the *clusterProfiler* R package [7]. *P* < 0.05 was considered statistically significant.

**Quantitative real-time polymerase chain reaction (qRT-PCR)**

RNA was extracted from bone marrow mononuclear cells using Trizol reagent (Life Technologies Corporation, Carlsbad, CA, USA) according to the instructions. 1μg of RNA sample was reverse transcribed to complementary DNA (cDNA) by using the 5×All-In-One RT MasterMix (ABM, Canada). 2×SYBR Green qPCR Master Mix (Bimake, USA) was used to examine the expression level of DLC1 (Primer: FW: GGACAGAGATGCCATTGAGGCT; RV: CACAAGGCTCATCCTCGTCTGA). The relative expression ratio of DLC1 was calculated by the 2-ΔΔCT method, and the median relative expression of DLC1 in all patients was used as the cut-off value between the high expression group and the low expression group.

**Statistical analysis**

All statistical analyses were performed with R software (version 3.6.1). WGCNA, ClusterProfiles, Forest plot, LASSO, SVM-RFE, and survival curves were plotted with R software. All statistical tests were two-sided, and *P* < 0.05 was considered statistically significant.

**Further** **discussion**

Our study applied a series of methods including WGCNA, LASSO, and SVM-RFE to analyze the GEO database, and screened five genes with potential prognostic value (DLC1, NF1B, DENND5B, TANC2, and ELAVL4). Survival analysis of the TCGA database and the data from our center showed that DLC1 is a valuable biological marker of AML, and the lack of DLC1 leads to poor prognosis.

DLC1, a tumor suppressor gene encoding a RhoGTPase-activating protein, is frequently downregulated or silenced in variety of solid tumors and hematological malignancies due to genomic deletions by epigenetic modifications [8, 9]. In several cancer types, reintroducing of DLC1 into tumor cells lacking endogenous gene expression inhibited cell proliferation, migration and invasion, and induced apoptosis in vitro. It can also reduce or prevent tumor formation and metastasis in nude mice [10]. A recent study confirmed DLC1 as a bona fide tumor suppressor gene using shRNA-mediated suppression of DLC1 expression in a murine liver tumor model. Mutations in the coding region of DLC1 are rare in human cancers, whereas homozygous or heterozygous deletions of DLC1 have been detected in some solid tumors. However, in liver, lung, colon, and breast tumors, the heterozygous deletions of DLC1 occurs more frequently than other tumor suppressor genes such as INK4/ARF, PTEN, or p53 [11]. Although DLC1 methylation was absent in normal bone marrow and lymphocytes, hypermethylation of DLC1 promoter has been detected in more than 80% of patients with acute lymphoblastic leukemia (ALL) and non-Hodgkin's lymphoma [12, 13]. Similar phenomenon was found in other hematological tumors, with hypermethylation of DLC1 was found in 78% of multiple myeloma patients and three multiple myeloma cell lines [14].

Previous studies have shown that DNA methylation is a dynamic process that affects the expression of related genes by altering the coding or non-coding sites. DNA methylation alterations in gene promoter regions have been shown to play a critical role in tumorigenesis. DLC1 is significant for the pathogenesis of ALL and its methylation inactivation is also associated to the prognosis of patients with leukemia. [15]. However, the role of DLC1 in AML has not been demonstrated in any reports. Our study showed that the reduction in DLC1 levels were more prominent in patients who died from AML. DLC1 deficiency is associated with poor long-term outcomes in AML patients. This may be due to the downregulation of DLC1 expression after DLC1 gene methylation modification in AML patients, which remains to be investigated.

Our study was based on bioinformatic library analysis and used a small number of AML samples for external validation, so the evidence is limited. The role and mechanism of DLC1 in AML need to be further verified by multi-center prospective studies with large sample size combined with in vivo and in vitro experiments.

1. **Additional Tables**

**Table S1. Clinical characteristics of 48 AML patients.**

| **Variables** | **Cases (%)** | **DLC1 expression level** | | ***p*** |
| --- | --- | --- | --- | --- |
|  |  | **High level (%)** | **Low level (%)** |  |
| **Gender** |  |  |  |  |
| Men | 28 (58.3) | 12 (50.0) | 16 (66.7) | 0.242 |
| Women | 20 (41.7) | 12 (50.0) | 8 (33.3) |  |
| **Age (years)** |  |  |  |  |
| <50 | 25 (52.1) | 13 (54.2) | 12 (50.0) | 0.773 |
| ≥50 | 23 (47.9) | 11 (45.8) | 12 (50.0) |  |
| **WBC (x10^9/L)** |  |  |  |  |
| <10 | 9 (18.8) | 3 (12.5) | 6 (25.0) | 0.460 |
| ≥10 | 39 (81.3) | 21 (87.5) | 18 (75.0) |  |
| **Hb (g/L)** |  |  |  |  |
| <90 | 34 (70.8) | 18 (75.0) | 16 (66.7) | 0.525 |
| ≥90 | 14 (29.2) | 6 (25.0) | 8 (33.3) |  |
| **PLT (x10^9/L)** |  |  |  |  |
| <100 | 40 (83.3) | 18 (75.0) | 22 (91.7) | 0.245 |
| ≥100 | 8 (16.7) | 6 (25.0) | 2 (8.3) |  |
| **Blasts in bone marrow** |  |  |  |  |
| <50% | 16 (33.3) | 9 (37.5) | 7 (29.2) | 0.540 |
| ≥50% | 32 (66.7) | 15 (62.5) | 17 (70.8) |  |
| **Karyotype** |  |  |  |  |
| Favorable | 6 (12.5) | 2 (8.3) | 4 (16.7) | 0.256 |
| Intermediate | 35 (72.9) | 20 (83.3) | 15 (62.5) |  |
| Adverse | 7 (14.6) | 2 (8.3) | 5 (20.8) |  |
| **Abbreviation:** WBC, white blood cell; Hb, hemoglobin; PLT, platelet. ***p*<0.01, **p*<0.05. | | | | |

| **Table S2. Basic information for the included GEO databases.** | | | | |
| --- | --- | --- | --- | --- |
| **Database No.** | **Authors** | **Sample size/Containing survival data** | **Median age (range, years)** | **Median follow-up (range, days)** |
| GSE37642 | Herold T et al | 562/422 | 57 (18-83) | 312.5 (1-5023) |
| GSE76009 | Ng SW et al | 534/307 | 52 (18-81) | 641 (43-5173) |
| GSE16432 | Bullinger et al | 436/436 | 49.96 (16.32-84.50) | 515 (1-3246) |
|  |  |  |  |  |

1. **Additional Figures**

**Additional figure legends**

**Figure S1. Correlation between gene significance and related module membership.**

A. The correlation between gene significance and module membership in the gray module. B. The correlation between gene significance and module membership in the blue module. **p* < 0.05, ***p* < 0.01, ****p* < 0.001.

**Figure S2. A Venn diagram shows the overlap of 22 optimal hub genes between GEO differential genes and WGCNA grey gene set.**

**Figure S3. Identification of candidate hub genes to predict prognosis for AML patients.**

A. Partial likelihood deviance for different numbers of variables revealed by the LASSO regression model. Red dots represent the partial likelihood deviance values. Gray lines represent the partial likelihood deviance ± standard error. B. Fourteen candidate genes with minimum lambda values were obtained by LASSO regression with tenfold cross-validation. C. Candidate genes were filtrated from SVM-RFE algorithms. D. Five optimal hub genes overlapped between LASSO and SVM-RFE algorithm were shown by Venn diagram. Abbreviations: LASSO, least absolute shrinkage and selection operator; SVM-RFE, support vector machine recursive feature elimination.

**Figure S4. The expression levels of DLC1, NFIB, DENND5B, TANC2, and ELAVL4 in AML patients with different survival status.**

Scatter plots of the five gene’s expression level in AML patients with different survival status based on GEO dataset. **p* < 0.05, ***p* < 0.01, ****p* < 0.001. Abbreviations: GEO, Gene Expression Omnibus.

**Figure S5. Kaplan-Meier survival curve according to the DLC1 expression level for 48 AML patients in our center.**

Kaplan-Meier plots was used to visualize the overall survival probability of 48 patients based on the expression of DLC1 at diagnosis in our center.

1. **Additional References**

1. Colaprico A, Silva TC, Olsen C, Garofano L, Cava C, Garolini D, Sabedot TS, Malta TM, Pagnotta SM, Castiglioni I *et al*: TCGAbiolinks: an R/Bioconductor package for integrative analysis of TCGA data. *Nucleic Acids Res* 2016, 44(8):e71.

2. Robinson MD, McCarthy DJ, Smyth GK: edgeR: a Bioconductor package for differential expression analysis of digital gene expression data. *Bioinformatics* 2010, 26(1):139-140.

3. Li T, Zhang Y, Patil P, Johnson WE: Overcoming the impacts of two-step batch effect correction on gene expression estimation and inference. *Biostatistics* 2021.

4. Davis S, Meltzer PS: GEOquery: a bridge between the Gene Expression Omnibus (GEO) and BioConductor. *Bioinformatics* 2007, 23(14):1846-1847.

5. Chen H, Boutros PC: VennDiagram: a package for the generation of highly-customizable Venn and Euler diagrams in R. *BMC Bioinformatics* 2011, 12:35.

6. Langfelder P, Horvath S: WGCNA: an R package for weighted correlation network analysis. *BMC Bioinformatics* 2008, 9:559.

7. Yu G, Wang LG, Han Y, He QY: clusterProfiler: an R package for comparing biological themes among gene clusters. *Omics* 2012, 16(5):284-287.

8. Carrasco DR, Tonon G, Huang Y, Zhang Y, Sinha R, Feng B, Stewart JP, Zhan F, Khatry D, Protopopova M *et al*: High-resolution genomic profiles define distinct clinico-pathogenetic subgroups of multiple myeloma patients. *Cancer Cell* 2006, 9(4):313-325.

9. Yuan BZ, Miller MJ, Keck CL, Zimonjic DB, Thorgeirsson SS, Popescu NC: Cloning, characterization, and chromosomal localization of a gene frequently deleted in human liver cancer (DLC-1) homologous to rat RhoGAP. *Cancer Res* 1998, 58(10):2196-2199.

10. Durkin ME, Yuan BZ, Zhou X, Zimonjic DB, Lowy DR, Thorgeirsson SS, Popescu NC: DLC-1:a Rho GTPase-activating protein and tumour suppressor. *J Cell Mol Med* 2007, 11(5):1185-1207.

11. Lahoz A, Hall A: DLC1: a significant GAP in the cancer genome. *Genes Dev* 2008, 22(13):1724-1730.

12. Shi H, Guo J, Duff DJ, Rahmatpanah F, Chitima-Matsiga R, Al-Kuhlani M, Taylor KH, Sjahputera O, Andreski M, Wooldridge JE *et al*: Discovery of novel epigenetic markers in non-Hodgkin's lymphoma. *Carcinogenesis* 2007, 28(1):60-70.

13. Pike BL, Greiner TC, Wang X, Weisenburger DD, Hsu YH, Renaud G, Wolfsberg TG, Kim M, Weisenberger DJ, Siegmund KD *et al*: DNA methylation profiles in diffuse large B-cell lymphoma and their relationship to gene expression status. *Leukemia* 2008, 22(5):1035-1043.

14. Song YF, Xu R, Zhang XH, Chen BB, Chen Q, Chen YM, Xie Y: High-frequency promoter hypermethylation of the deleted in liver cancer-1 gene in multiple myeloma. *J Clin Pathol* 2006, 59(9):947-951.

15. Rahmani M, Talebi M, Hagh MF, Feizi AAH, Solali S: Aberrant DNA methylation of key genes and Acute Lymphoblastic Leukemia. *Biomed Pharmacother* 2018, 97:1493-1500.
